# FINET: Fast Inferring NETwork

**DOI:** 10.1186/s13104-020-05371-0

**Published:** 2020-11-10

**Authors:** Anyou Wang, Rong Hai

**Affiliations:** 1 The Institute for Integrative Genome Biology, University of California at Riverside, Riverside, CA 92521 USA; 2 Department of Microbiology and Plant Pathology, University of California at Riverside, Riverside, CA 92521 USA

**Keywords:** FINET, Network, Inference, Julia, Stability selection, Elastic-net, LASSO, Accuracy

## Abstract

**Objectives:**

Numerous software has been developed to infer the gene regulatory network, a long-standing key topic in biology and computational biology. Yet the slowness and inaccuracy inherited in current software hamper their applications to the increasing massive data. Here, we develop a software, FINET (Fast Inferring NETwork), to infer a network with high accuracy and rapidity from big data.

**Results:**

The high accuracy results from integrating algorithms with stability-selection, elastic-net, and parameter optimization. Tested by a known biological network, FINET infers interactions with over 94% precision. The high speed comes from partnering parallel computations implemented with Julia, a new compiled language that runs much faster than existing languages used in the current software, such as R, Python, and MATLAB. Regardless of FINET’s implementations with Julia, users with no background in the language or computer science can easily operate it, with only a user-friendly single command line. In addition, FINET can infer other networks such as chemical networks and social networks. Overall, FINET provides a confident way to efficiently and accurately infer any type of network for any scale of data.

## Introduction

All biological phenotypes are achieved from fine regulation of gene expression. Thus, understanding gene regulations is a crucially fundamental topic in the biology. Conventionally, manipulating gene mutations such as knockout and knockdown helps to digest the gene regulations. However, these approaches suffer several drawbacks such as transcript compensatory and side effects [1]. Gene mutation approaches also assume that the genome remains stable after mutations. However, the genome varies dramatically with even a single gene mutation, which alters gene expressions of thousand genes as shown in RNA sequencing data. As a result, there is no way to fully comprehend the complete regulatory interactions of any single gene.

Computational biology and bioinformatics have attempted to infer gene regulatory networks from gene expression data, and have established software and tools to execute their works [2–9]. However, the efficiency of current software suffers from high noise and lagging. They usually generate overly complicated network interactions—mostly false positives [2]. Therefore, these results actually provide more questions than answers to true biology regulatory interactions. In addition, the current software face challenges when applied to big sequencing data. With the software FINET, we are able to quickly and accurately reveal true gene interactions and refresh gene interaction pictures from massive data.

## Main text

### Theory and algorithms

Theoretically, FINET is based on elastic-net theory and stability selections. The elastic-net is an extension of LASSO [10] (least absolute shrinkage and selection operator, referring to theory and algorithm [11] for detailed), a penalized regression method for shrinkage and variable selection by minimizing:

$$\sum\limits_{i = 1}^{n} {\left( {y_{i} - \sum\limits_{j} {x_{ij} }\upbeta_{j} } \right)}^{2} + \lambda \sum\limits_{j = 1}^{p} {\left| {\upbeta_{j} } \right|}$$

*i* = 1, 2, …, *n* (*n* equivalent to sample size); *j* = 1, 2, …, *p* (*p* equivalent to omics gene number); *y*_*i*_ = response variable of sample *i*, β_*j*_ = coefficient foe gene *j*, *j* = 1, 2, …, *p*, and *x*_*ij*_ = observation value of sample *i* and gene *j*.

Lasso tends to ignore the variables in a correlated group. To include the correlated genes, the elastic-net adds an additional quadratic part $\sum\nolimits_{j}{\beta}_{j}^{2}\le t$ to the penalization.

Elastic-net and lasso are arguably the best methods for shrinkage and variable selection, and k-fold cross-validations have been implemented in current software like GMLNet [12]. However, these validations include too many variables and these selected variables offer results of coefficients without any priority of trueness. It is then difficult to estimate the stability of these variable selections.

To improve the accuracy of variable selection, stability selection comes into play [13]. The general idea of stability selection is to add a re-sampling step into an existing model selection to make it stable and increase accuracy. For example, during elastic-net selection, the total samples are randomly partitioned into two subgroups, and each subgroup is subjected to an elastic-net model selection. If a variable was simultaneously selected at the two groups, the selected variable would be likely true [13].

The FINET’s algorithm of each resampling step is to bootstrap randomly split samples into m subgroups (m ≥ 2) without replacement. In each subgroup, a complete model of elastic-net is run to select variables (regulators in biology) interacting with a target (a target gene in biology). Such resampling step iterates *n* times. The frequency of each regulator selected during iterations is counted as frequency score. Frequency score is equal to total selected times in n*m trials (total hits/n*m), and it is used to rank regulator priority of confidences (frequency levels) and confidence strength in true positive selection. The maximum frequency score is 1 (the highest confidence). A variable with a frequency score of 1 for a given target means that it was always selected in m*n trials and it is likely a true positive regulator for this target. When m increases (e.g. m = 8), in which a regulator simultaneously targets its target at m sub-groups in n bootstrap resampling, type I error goes down dramatically.

### Parameter optimization

We have optimized FINET parameters for most common users and these parameters were set as default values in FINET. Here, we only highlighted parameter optimization of the frequency score cutoff and resampling in *m* groups.

### Frequency score cutoff

To systematically optimize the frequency score cutoff for FINET, we run FINET to select regulators controlling each target in a well-known matrix established by dream5 network challenge, network1 [2] (Table 1), which includes an in silico matrix (1643 genes and 805 observations) and golden standard true positives derived from well-established regulatory database, regulonDB [14].

**Table 1:** Test dataset features

Source	Dream5 network1
Data type	In silico
Structure of interactions	RegulonDB
Size (observation*genes)	805*1643
Total interactions	278,392
True interactions	4012
False interactions	274,380

**Fig. 1:**
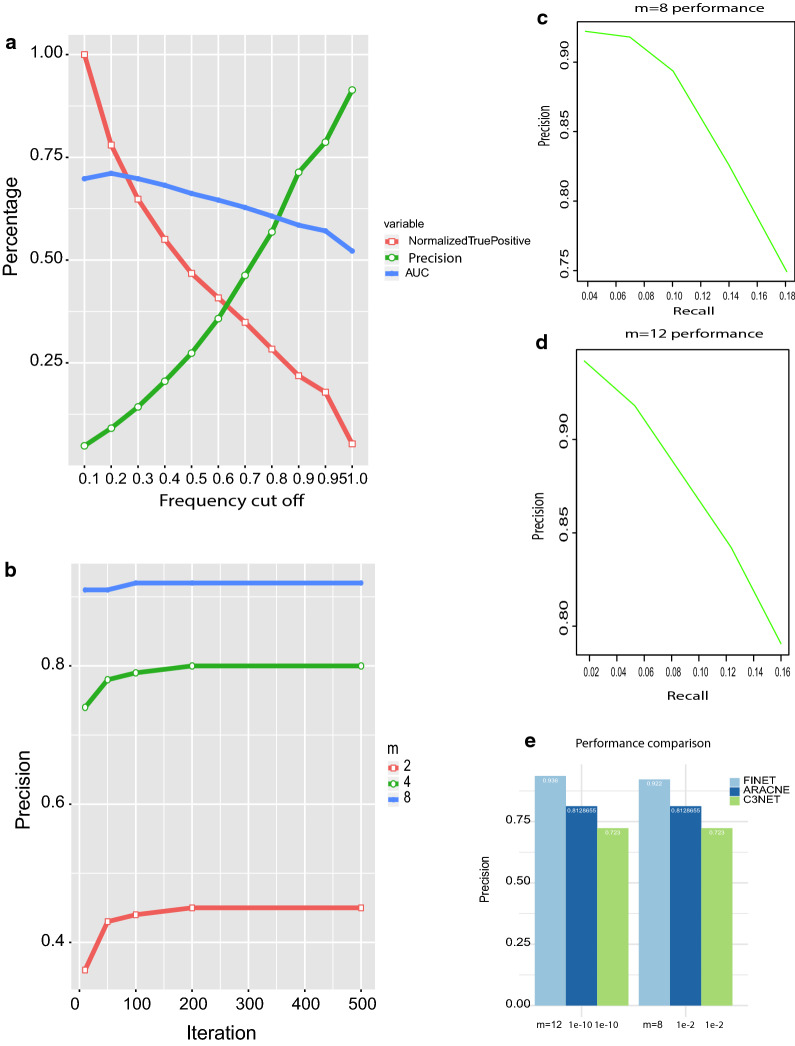
FINET parameter optimization and performance. **a** Frequency cutoff optimization. Frequency cutoff from 0.1 to 1.0 vs AUC, precision and normalized true positive calling (true positive callings at each cutoff/max(true positive callings at each cutoff)). This data resulted from FINET running on network1 at dream5 with following settings, m = 4, n = 500, alpha = 0.5 (see github software website for details). **b** Comparisons of precision of resampling m subgroups (frequency cutoff > 0.95). **c**, **d** The overall performance of FINET when m = 8 (**c**) and 12 (**d**). **e** Performance comparison between FINET, ARACNe-AP and C3NET. X-axis lab fo ARACNe-AP and C3NET represent p-value and alpha value, respectively, designed for significant threshold in ARACNe-AP and C3NET, while m in FINET as the number of sub-groups as shown above

From the theory above, we learned that a high frequency cutoff ensures the accuracy of variable selection. The optimal cutoff, however, remains unknown. To optimize the frequency cutoff, we first computed the AUC (Area Under The Curve) of ROC (receiver operating characteristic curve) at an array of frequency from 0.1 to 1. The golden standard at network1 was treated as known interactions, and the total true positives produced by FINET were treated as true positive callings, and the rest were negative callings. As expected, the AUC decreased with increasing frequency cutoff (Fig. 1a, blue line). At the frequency cutoff of 0.2, AUC reached 71.1%, but at the frequency cutoff of 0.95, the AUC lowered to 57.1%. This was consistent with the trend of total true positive callings, which declined dramatically with a high frequency cutoff (Fig. 1a, red line). Obviously, at lower frequency cutoff, more positives were selected and less negatives were filled in. This resulted in higher AUC, but it contained higher noise because more false positives were also added to the selection. Therefore, AUC may not be a good measurement to evaluate the accuracy of true positive calling.

Here, we used precision (true positives/total true positive callings) to measure accuracy. During variable selection, we normally select too many variables, unsure of which one is true. In the network inference, it is more meaningful to have a higher precision than to call more true positives including noise. In fact, some interactions in biology may not be relevant or condition-dependent, and ignoring some interactions might make the network clear. Many biological experiments are normally conducted to prove one true gene interaction. It is valuable to obtain real true positives from computational biology. Adding false positives to get the high AUC would jeopardize the scientific value of findings. Therefore, the precision has more advantage than AUC.

The precision increased positively with frequency cutoff (Fig. 1a green line). When frequency cutoff at 0.95, the precision reached 80% at resampling m = 4. A higher frequency cutoff directly correlated to a higher precision and inversely related to the error ratio. These results fit the theory above very well. In contrast, more than 90% of true positive callings were false positives at cutoff = 0.1, indicating most selections (> 90%) as false without stability-selection resampling step. Therefore, the high frequency cutoff (e.g. 0.95) reduces false positive callings and makes selection stable and robust. Stability-selection resampling is necessary and important for selecting correct variables.

### Resampling m subgroups

Resampling is the key technique to improve the precision in FINET, which allows resampling m subgroups. We plotted the precision for each m (m = 2,4,8) and evaluated the effect of m on the precision. When m = 2, the maximum of precision only reached 45% at n = 200 iterations and still kept a lot of noise, although m = 2 was proposed and adopted in most current software [2, 13].

To solve the high noise problem, FINET increases m value as described above. FINET reaches 80% and 92% for m = 4 and 8 respectively (Fig. 1b). In addition, when m = 8, the precision reached 91% with n = 10 iterations, and only slightly increased to 92% at n = 100. Precision became stable at n = 200. Therefore, increasing iteration n value to a big number like 10,000 as suggested in most software might not help a lot.

To appreciate the overall improvement from FINET, we plotted its precision against recall for m = 8 and m = 12 (Fig. 1c, d). When m = 8 and frequency cutoff with 0.99, 0.95, and 0,9, the precision of FINET reaches respectively 92.2%, 91.8%, and 89.4% with recall 0.04, 0.07, 0.1 (Fig. 1c). Increasing m to 12 improves precision to 94.2%, 93.6%, 91.8% respectively for frequency cutoff of 0.99, 0.95 and 0.9, with recall 0.02, 0.04, 0.05 (Fig. 1d). This suggested that the best way to improve accuracy is to increase sample size to allow big m value (e.g. m ≥ 8).

### Performance comparison

To compare the performance of FINET to other existing software, we compared it to C3NET and ARACNe-AP that were reported as top performers in network inferences [8, 9]. We still used the dream network1 to calculate the precision obtained by both FINET, C3NET and ARACNe-AP. FINET precision increased from 92.2 to 94.2% when m values changed from 8 to 12 as described above, and obviously FINET could go beyond 94.2% if m increased to 14 or 16 when sample size is available. In contrast, C3NET and ARACNe-AP only got 72.3% and 81% precision respectively when the statistical significance threshold (alpha value set by C3NET) was set from 0.01 to 1e−10 (Fig. 1e). Actually, bother C3NET and ARACNe-AP was not sensitive to cutoff (alpha in C3NET and *p* value in ARACNe-AP), but ARACNe-AP responded to bootstrap number. ARACNe-AP could reach the highest precision (0.81 at 5 reproducible bootstraps) but its precision declined with more bootstraps (e.g. precision of 0.52 at 20 reproducible bootstraps). However, FINET was very sensitive to parameter settings as discussed above. FINET designs to get high precision.

### Implementation, speed and usage

To get high precision, FINET employs sophisticated algorithms including Elastic-net. Elastic-net has a computational complexity close to O(n^3^) when variables > observations [15] although the complexity varies with implementations. This high complexity leads to slowness in computation. To solve the slowness problem while keeping high precision performance, we implemented FINET with parallel computations in Julia, a new language with speed comparable to C/C++. From julia 0.4 to its latest version, we believe the multiple process as the stable module for parallel computations in Julia, although other approaches have been introduced. Therefore, FINET still uses multiple process modules for parallel computations. Running multiple processes requires big memory for large quantities of data. This issue is solved by using shared arrays across the processes to reduce the memory consumption in FINET.

The speed of FINET develops on many parameters, including variables and user customer settings such as CPU number, iterations (n), sub-groups in stability-selection (m), k validations in elastic-net model. Therefore, it is hard to find reasonable metrics to compare its direct speed with other software. It seemed reasonable to compare the time for a same process. For example, comparing the same Fortran code of elastic-net model, glmnet, running respectively in R and Julia for a random matrix 10,000*100, Julia and R took respectively 0.7541 and 1.166 s to complete a single cross-validation fit. This is expected because it is known that Julia run much faster than R, Python and MATLAB, which are widely used in network inference software. However, this did not mean that FINET always completes a network inference faster than other software because a single process is only the core process to select variables and FINET has high complexity inside its math models and algorithms as described above. In another way, we can measure the run-time of completing a task at a given condition. Here, we compared FINET, C3NET and ARACNe-AP in a single computer node with 40 CPUs by using network1 in dream5. FINET, C3NET and ARACNe-AP completed it with 108.692079618, 82.727504605 and 145.465978563 s respectively (Table 2). This should represent the computational complexity of these three software but FINET could go faster than that if more CPUs were available. Again, FINET speed develops on user settings.

**Table 2:** Comparison of FINET and C3NET

Metrics	FINET	ARACNe-AP	C3NET
Precision (%)	94.2	0.81	72.3
Parameter sensitivity	Very	Moderate	Not
Implementation	Julia	Java	R
Big data	Fast	Moderate	Not practical
Sample size	Unlimited	65 k	Not practical
Gene size	Unlimited	Limited	Not practical
Complete net1 (s)^a^	108.6920796	145.4659786	82.7275046
Memory	Share	Thread share	Not

For comparable speed test of all software, FINET parameters were set to simple settings, m = 1 with 5 cross-validations, and ARACNe was set to 5 bootstraps

^a^Completed in a computer node with 40 CPUs. All genes were used to infer their interactions

For the big data, it is unpractical to use C3NET to run big data like a 100,000*100,000 matrix due to its single CPU structure in slow R environment. ARACNe-AP implementation with parallel computation can run up to 65 536 samples and a limited gene list [16]. FINET designs for the big data with scalable properties in parallel computations and shared memory management.

Using FINET is easy. FINET completes all processes with one simple command line, with input data and output file names as required, and other arguments as optional and default. The input data is a normalized matrix with each column as a gene and rows as observations (see the github web for details). Anyone with or without a computer science background can easily complete the command line.

Although developed under Linux environment, FINET should perform well in any operating system with Julia installation, including microsoftware window and apple machintosh.

## Conclusion

This study developed algorithms and software, FINET, to infer network with both high accuracy and speed. Due to its scalability in parallel computation, FINET is specifically useful for big data analysis.

### Limitation

This software should not be applied to unnormalized data at current stage until further development note.

## Data Availability

Availability and implementation available in github https://github.com/anyouwang/finet.git. Application samples shown in our manuscript titled “Big-data analysis unearths the general regulatory regime in normal human genome and cancer” 10.1101/791970. Detailed application data https://combai.org/network/.

## Abbreviations

FINET: Fast Inferring NETwork
LASSO: Least absolute shrinkage and selection operator
AUC: Area Under The Curve
ROC: Receiver operating characteristic curve
